# Group Theory of Syntactical Freedom in DNA Transcription and Genome Decoding

**DOI:** 10.3390/cimb44040095

**Published:** 2022-03-22

**Authors:** Michel Planat, Marcelo M. Amaral, Fang Fang, David Chester, Raymond Aschheim, Klee Irwin

**Affiliations:** 1 Institut FEMTO-ST CNRS UMR 6174, Université de Bourgogne-Franche-Comté, F-25044 Besançon, France; 2 Quantum Gravity Research, Los Angeles, CA 90290, USA; marcelo@QuantumGravityResearch.org (M.M.A.); fang@QuantumGravityResearch.org (F.F.); davidc@QuantumGravityResearch.org (D.C.); raymond@QuantumGravityResearch.org (R.A.); klee@QuantumGravityResearch.org (K.I.)

**Keywords:** DNA transcription factors, finitely generated groups, aperiodic order

## Abstract

Transcription factors (TFs) are proteins that recognize specific DNA fragments in order to decode the genome and ensure its optimal functioning. TFs work at the local and global scales by specifying cell type, cell growth and death, cell migration, organization and timely tasks. We investigate the structure of DNA-binding motifs with the theory of finitely generated groups. The DNA ‘word’ in the binding domain—the motif—may be seen as the generator of a finitely generated group $F_{dna}$ on four letters, the bases A, T, G and C. It is shown that, most of the time, the DNA-binding motifs have subgroup structures close to free groups of rank three or less, a property that we call ‘syntactical freedom’. Such a property is associated with the aperiodicity of the motif when it is seen as a substitution sequence. Examples are provided for the major families of TFs, such as leucine zipper factors, zinc finger factors, homeo-domain factors, etc. We also discuss the exceptions to the existence of such DNA syntactical rules and their functional roles. This includes the TATA box in the promoter region of some genes, the single-nucleotide markers (SNP) and the motifs of some genes of ubiquitous roles in transcription and regulation.

## 1. Introduction

In his recent paper, Klee Irwin writes “*Reality would be non-deterministic, not because it is random, but because it is a code—a finite set of irreducible symbols and syntactical rules. 0ur definition of information is meaning conveyed by symbolism. And expressions of code or language are strings of symbols allowed by syntax—ordering rules with syntactical freedom.*” [1].

Our last papers focused on the relevance of free groups in the encoding of the secondary structure of proteins [2] and in the encoding of tonal music and poetry [3].

In this present contribution, the concept of syntactical freedom is associated with that of a free group in the encoding of strings of symbols—the motifs of DNA transcription. It is shown for the first time that most transcription factors, but not all, have motifs where the DNA letters in the motif form a finitely generated group whose structure is close to a free group. The exceptions rely either on a specific functional role of the DNA sequence under investigation or a potential dysfunction in the transcription of the gene, resulting in disease.

A few definitions in the domain of genetics that we use in the paper are as follows:

**Sequence motif** An amino-acid sequence pattern that is related to a biological function or a gene. The motif is sometimes called a ‘consensus sequence’.

**DNA-binding domain** A folded protein domain that contains a structural motif that recognizes double- or single-stranded DNA.

**Transcription factor** A sequence-specific DNA-binding factor, or transcription factor, is a protein that controls the rate of transcription of a gene from DNA to messenger RNA by binding to a specific DNA sequence. There are approximately 1600 binding domains in the human genome that function as transcription factors. Classes of DNA-binding domains of transcription factors also exist. The most common are zinc-coordinating DNA-binding domains, helix-loop-helix or helix-turn-helix motifs, basic leucine zipper domains and homeobox domains (playing critical roles in the regulation of development). A classification of human transcription factors and their structural motifs is in References [4,5,6].

**Exon** A part of a gene that encodes a part of the mature RNA produced by that gene after removing all introns (the non-coding regions of RNA transcript) by RNA splicing.

**Promoter** A sequence of DNA in which proteins initiate the transcription of a single RNA from the DNA downstream of it. The TATA box is a sequence found in the core promoter region of some genes in archaea and eukaryotes.

**Zinc finger** A small protein structural motif containing one or more zinc ions in order to stabilize the protein fold.

**Protein** isoform A set of highly similar proteins may originate from a single gene. This process is regulated by the alternative splicing of mRNA. In this process, particular exons of a gene may be included within or excluded from the final, processed messenger RNA (mRNA) produced from that gene. Alternative splicing and the multi-exonic genes are a common feature in eukaryotes.

## 2. Materials and Methods

### 2.1. Finitely Generated Groups

In the domain of algebra, a group is a set equipped with an operation between every pair of elements of the set that is associative, has an identity element, and every element has an inverse. The elements of a group may be numbers or other structures.

One familiar group is the set Z of relative integers, together with the addition as the group law. Notice that Z has infinite elements. The addition in Z is commutative, not a general property for other groups.

The groups under consideration in this paper have elements that are words on four letters A, T, G and C—the bases in DNA—and the group law is the product. In the context of transcription factors (TFs), a motif fully describes the group. For example, taking only two letters/bases A and T and a relation/motif rel(A,T) = TATA, the group is of the finitely generated type and the mathematical notation is $f_{p}=\langle A,T|TATA=I\rangle$, in which the motif equals the identity element *I*. Infinite elements exist in fp. The group fp is non-commutative and contains a wealth of symmetries. One useful method for investigating them consists of two important concepts.

The first one is the concept of a free group on two generators $F_{2}=\langle A,T\rangle$ with no explicit relation (but not forgetting the relations following from the axioms of a group: $AA^{-1}=TT^{-1}=I$). The second concept is that of a conjugacy class of subgroups. Two elements *a* and *b* belong to the same class if they are conjugate, meaning that *a* and $b=g^{-1}ag$ belong to the same class for every g∈fp.

The finitely generated group with the relation rel(A,T) = TATA may be characterized by the number of conjugacy classes at each index d∈[1,2,3,…] with the sequence $\eta_{d}=\left[1,3,3,10,15,56,131,462,\ldots \right]$. A signature of the group is also shared by the group of ‘dessins d’enfants’ in [7]—which the reader may consult for an introduction to the field of finitely generated groups and the related graphs, topology and geometry.

### 2.2. Free Groups and Their Conjugacy Classes

Let $F_{r}$ be the free group on *r* generators. Following a theorem derived by Hall in 1949 [8], the number $N_{d,r}$ of subgroups of index *d* in $F_{r}$ is

$$N_{d,r}=d{\left(d!\right)}^{r-1}-\sum_{i=1}^{d-1}{\left[(d-i)!\right]}^{r-1}N_{i,r}$$

leading to the number Isoc(X;d) of connected *d*-fold coverings of a graph *X* (i.e., the number of conjugacy classes of subgroups in the fundamental group of *X*) is as follows [9] (Theorem 3.2, p. 84),

$$\mathrm{Isoc}\left(X;d\right)=\frac{1}{d}\sum_{m|d}N_{m,r}\sum_{l|\frac{d}{m}}\mu \left(\frac{d}{ml}\right)l^{(r-1)m+1},$$

where μ denotes the number-theoretic Möbius function.

Table 1 provides the values of Isoc(X;d) for small values of *r* and *d*, see [2] and ([9] [Table 3.2]).

We are interested in the cardinality sequence (card seq) of conjugacy classes for subgroups of a finitely generated group fp with a relation (rel) given by the sequence motif. Most of the time, the DNA motif in the transcription factor is close to that of a free group $F_{r}$, with r+1 being the number of distinct bases involved in the motif. However, the finitely generated group $f_{p}=\langle x_{1},x_{2}|rel\left(x_{1},x_{2}\right)\rangle$, or $f_{p}=\langle x_{1},x_{2},x_{3}|rel\left(x_{1},x_{2},x_{3}\right)\rangle$, or $f_{p}=\langle x_{1},x_{2},x_{3},x_{4}|rel\left(x_{1},x_{2},x_{3},x_{4}\right)\rangle$ (where the $x_{i}$ are taken in the four bases A, T, G, C and rel is the motif) is not the free group $F_{1}=\langle x_{1},x_{2}|x_{1}x_{2}\rangle$, or $F_{2}=\langle x_{1},x_{2},x_{3}|x_{1}x_{2}x_{3}\rangle$, or $F_{3}=\langle x_{1},x_{2},x_{3},x_{4}|x_{1}x_{2}x_{3}x_{4}\rangle$. The closeness of $f_{p}$ to $F_{r}$ can be checked in the finite range of indices of the card seq.

### 2.3. Content of the Paper

The structure of the TATA box in the core promoter region of many eukaryotes is not close to that of a free group. Remarkably, the card seq of $f_{p}$ for the TATA box is close to that of the Hecke group $H_{q}=\langle x_{1},x_{2}|x_{1}^{2}=x_{2}^{q}\rangle$ [10]. The case q=3 corresponds to the modular group PSL(2,Z), which is the fundamental group of the trefoil knot manifold $K3a1=3_{1}$. The Hecke group $H_{q}$, with q≥3, is the discrete group generated by z→−1/z, $z\to z+\lambda_{q}$, where $\lambda_{q}=2\cos\left(\pi /q\right)$ with $\lambda_{3}=1$, $\lambda_{4}=\sqrt{2}$, $\lambda_{5}=\left(1+\sqrt{5}\right)/2$, $\lambda_{6}=\sqrt{3}$, etc. In Section 3.1, it is shown that the card seq for motifs in the standard TATA box corresponds to $H_{3}$ or $H_{4}$ and that, in the case of a Gilbert’s syndrome, it is only approximate or corresponds to $H_{q}$, q>4.

In the same section, we investigate single-nucleotide polymorphisms (SNPs) of some genes. In the case of SNP markers involving 3 bases, the fit of the card seq to that of the free group $F_{2}$ is obtained, or not. The fit of the card seq of the selected SNP to that of $F_{2}$ is well correlated to a lower risk of disease.

In Section 3.4, we analyze the binding domains and the card seq associated with motifs of the immediately early genes Fos, EGR1 and Myc. In such cases, the card seq of the group fp, taken with the relation as the motif, is that of a free group $F_{r}$ (in the finite range of indices). Most of the time, the motif of a transcription factor for a gene leads to the card seq of a $F_{r}$. This statement follows from an almost exhaustive investigation of transcription factors found in the databases defined in References [4,6].

However, it is also important to investigate the transcription factors with a group structure away from a free group. This is done in Section 3.5 with the claim that the lack of syntactical freedom (i.e., that the card seq of the gene is not that of a free group) is a marker of potential dysfunction of the gene through mutations or isoforms.

In Section 4, we show that group theoretical freedom correlates to the aperiodicity of motifs when the latter are seen as substitution sequences.

In the Conclusions, we offer some roads of progress in the connection of group theory to genetics.

## 3. Results

### 3.1. The TATA Box, the Hecke Groups and More

The TATA box (also called the Goldberg–Hogness box) is a DNA sequence located in the core promoter region of genes in many eukaryotes, as well in archaea [11]. The TATA box is a non-coding sequence whose name comes from the fact that it contains a consensus sequence with repeating T and A base pairs [12,13]. The TATA box is a component of eukaryotic promoters in which it initiates the transcription of TATA-containing genes. The TATA box binds to the TATA-binding protein (TBP) and some other transcription factors. TBP binds to the minor groove of the TATA box via a region of antiparallel β sheets in the protein.

The regulation of gene transcription by transcription factors depends on the gene and is governed by the RNA polymerase II (PolII) transcription complex. In the core promoter of a typical PolII, they are key elements such as a TATA box.

Mutations such as insertions, deletions and point mutations to this consensus sequence can result in phenotypic changes. These changes can then be related to diseases such as gastric cancer, blindness, immunosuppression, Gilbert’s syndrome, etc. [12].

### 3.2. Gilbert’s Syndrome

Gilbert’s syndrome is a genetic polymorphism associated with the gene UGTIA1, a phase II drug-metabolizing enzyme, which is essential in the metabolism of bilirubin and other drugs. The core promoter in UGTIA1 contains a TATA box located at position −28 with respect to the transcriptional start site [14]. A polymorphism with AT(TA)${}_{l}$TAA (with l = 5 … 8) is common in all ethnic populations with *l* = 6 as the major allele. Minor alleles with l>6 have less UGTIA1 transcription efficiency, leading to Gilbert’s syndrome, neonatal jaundice and toxicity in cancer chemotherapy [14].

In Table 2, we look at the finitely generated groups $fp=\langle A,T|rel\rangle$, where rel is the consensus sequence in the TATA box. The first two rows are for a standard TATA box. For this case, the group fp is found to have the same cardinality structure of cc of subgroups as the group $H_{3}$ (the modular group) or the Hecke group $H_{4}$. Rows 3 and 4 are for the TATA box in the core promoter of the UGTIA1 gene for normal subjects, while rows 7 and 8 are for subjects with a Gilbert’s syndrome. In the former case, the group fp has a cardinality structure of cc of subgroups corresponding to the Hecke groups $H_{6}$ and $H_{7}$, while in the latter case, the cardinality structure of cc of subgroups fits that of the Hecke groups $H_{4}$ and $H_{3}$ only up to index 8. Thus, we find that Gilbert’s syndrome is associated with an imperfect fit of the group *G* to a Hecke group.

### 3.3. Single Nucleotide Polymorphism

The canonical form of TBP-binding sites, the TATA box, is the best-studied regulatory element among human gene promoters. Tables identifying single-nucleotide polymorphisms (SNPs) in the (gene-dependent) TATA box have been collected in Reference [15].

At present, there are approximately $10^{8}$ stored SNP markers that have been identified in the human genome and approximately $10^{10}$ potentially possible markers. Most of them are neutral and do not affect health in any way. Markers in protein-coding regions of genes may damage proteins but are uncorrectable by treatment or lifestyle changes. However, a large number of the variants that have been identified are located in non-protein-coding regions and are presumed to affect gene expression regulation [16]. Regulatory SNPs in the TATA regions have biomedical usefulness and are correctable by medication and/or lifestyle. Ref. [15] collects 126 known SNP markers in 7 tables. We made use of these tables to compute the finitely generated group fp whose relation (rel) is the marker; see Table 3. For simplicity, we only took SNP markers built from 3 bases (and the exceptional SNP marker with 2 bases). We made the cardinality sequence of cc of subgroups (card seq) explicit. The computed closeness of the finitely generated group to the free group $F_{2}$ correlates to a lower risk of illness. On the contrary, markers leading to a card seq away from that of $F_{2}$ indicate a potential higher risk of illness. The symbol * corresponds to the only two-base SNP marker in the table. In this case, the card seq is the same as the sequence for the fundamental group of 3-manifold $m_{002}=net02_{00000}$. The latter manifold is the smallest volume closed 3-manifold and is non orientable [17].

As a way of example, we take SNP markers in the first section of Table 3 that correspond to potential tumors in reproductive organs. Five of them show a card seq away from that of the free group $F_{2}$, and they also correspond to a potential higher risk of disease. The last two markers in the same section, whose card seq is close to that of $F_{2}$, are expected to produce a lower risk of breast cancer. Similar conclusions are valid for the SNP markers in other sections of Table 3.

### 3.4. A Few DNA/Protein Complexes and Their Transcription Factors

As mentioned in the Introduction, most transcription factors have a binding domain whose finitely generated group fp has a subgroup signature equal to that of a free group $F_{r}$ of rank *r*, with r≤3 corresponding to r+1≤4 distinct letters. An almost exhaustive search has been performed by using the catalogs found in [4,6]. We first give details of our calculations for a few immediate early genes. Then, in Section 3.5, most found counterexamples are summarized.

#### 3.4.1. Immediate Early Genes and Their Motifs

Immediate early genes (IEGs) are genes that are activated transiently and rapidly in response to a wide variety of cellular stimuli. They represent a standing response mechanism that is activated at the transcription level in the first round of response to stimuli, before any new proteins are synthesized. The earliest known and best characterized include c-fos, c-myc and c-jun, genes that were found to be homologous to retroviral oncogenes. IEGs are well known as early regulators of cell growth and differentiation signals. However, other findings suggest roles for IEGs in many other cellular processes as ‘gateways to genomic response’. Many IEG products are natural transcription factors or other DNA-binding proteins. Important classes of IEG products include secreted proteins, cytoskeletal proteins and receptor subunits.

Some IEGs such as ZNF268 and Arc have been implicated in learning, memory and long-term potentiation. Neuronal IEGs are used prevalently as a marker to track brain activities in the context of memory formation and the development of psychiatric disorders [18].

The group structure of the motifs of some IEGs in the Fos, EGR and Myc classes is summarized in Table 4.

#### 3.4.2. The DNA-Binding Domain Fos

**Figure 1:**
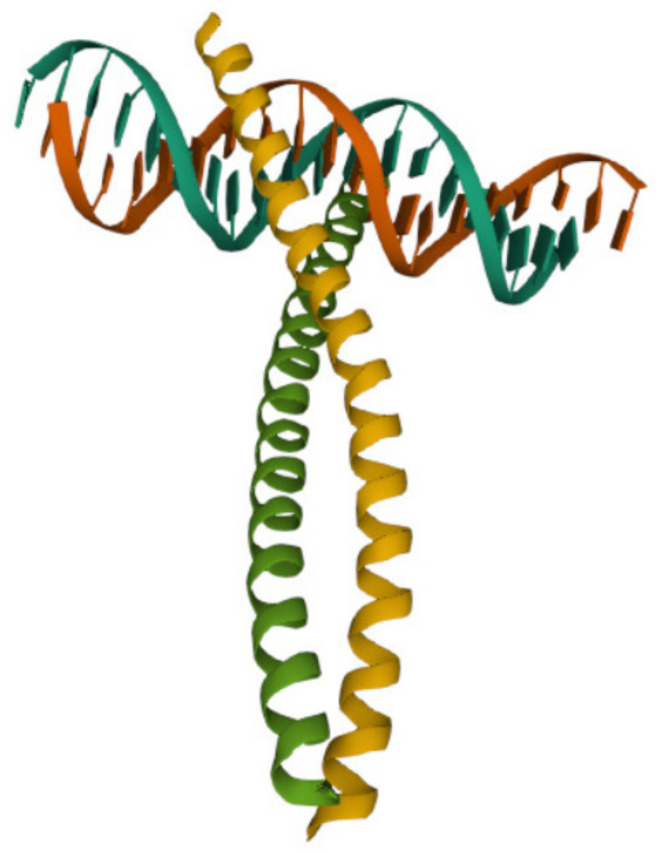
The DNA-binding domain of the immediate early gene Fos. The name in the protein data bank is 1FOS.

The Fos family (as well as the Jun family) are eukaryotic transcription factors that heterodimerize to form complexes binding elements such as 5${}^{\prime }$-TGAGTCA-3${}^{\prime }$ DNA elements [19]. The X-ray crystal structure was determined and the bZIP region (with 62 aa) of the c-Fos protein bound to DNA is available in the protein data bank as PDB: 1FOS. The protein secondary structure of this subunit of c-Fos protein consists of two alpha helices, as shown in Figure 1.

Let us consider the group $fp=\langle A,T,G,C|rel)\rangle$ on 4 letters with the relation rel=bind=TGAGTCA. The card seq of $f_{p}$ up to index 6 is that of the free group $F_{3}=\langle A,T,G,C|\mathrm{AGTC})\rangle$ of rank 3. One can use the coset enumeration (with the Todd–Coxeter procedure) to check that, up to index 6, the permutation groups organizing the cosets in the cc of groups $f_{p}$ and $F_{3}$ are the same. This shows that both groups are close, at least in the finite range of subgroups. However, fp and $F_{3}$ are not the same group. Incidentally, the group $fp^{\prime }=\langle A,T,G,C|\mathrm{AGTC},\mathrm{bind})\rangle$, with the joint relations of fp and $F_{3}$, is close to the free group $F_{2}=\langle x,y,z|xyz\rangle$ on two generators in the sense that the cardinality sequence of the cc of subgroups is that of $F_{2}$, up to the higher index 9 that we could reach in our calculations.

Similarly, the finitely generated groups $fp=\langle A,T,G,C|\mathrm{rel}\rangle$, where the relation is with the whole DNA chains rel = AATGGATGAGTCATAGGAGA (1FOS_1) or rel = TTCTCCTATGACTCATCCAT (1FOS_2) involved in the DNA/protein Fos complex (PDB:1F0S), have the same card seq as $F_{3}$ up to index 6.

#### 3.4.3. The DNA-Binding Domain EGR1

**Figure 2:**
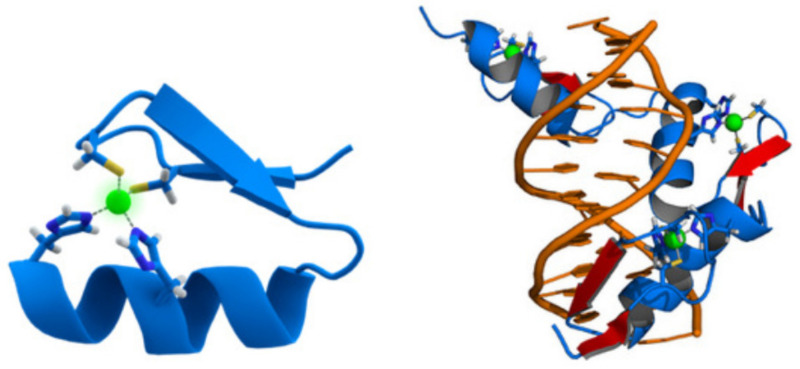
(**Left**) Cartoon representation of the Cys${}_{2}$His${}_{2}$ zinc finger motif, consisting of an α-helix and an antiparallel β-sheet. The zinc ion (green) is coordinated by two histidine residues and two cysteine residues. (**Right**) Cartoon representation of the protein ZNF268 (blue) containing three zinc fingers in complex with DNA (orange). The coordinating amino acid residues and zinc ions (green) are highlighted. The name of the DNA-binding domain in the protein data bank is 4R2A.

The DNA-binding domain EGR1 (for early growth response protein 1) is a mammalian transcription factor also called ZNF268 (the zinc finger protein 268). This is because the protein encoded by the EGR1 gene has the Cys${}_{2}$His${}_{2}$-like fold structure of a zinc finger, as shown in Figure 2. It binds to the motif $5^{\prime }$-bind-$3^{\prime }$ [20], with bind = GCG(T/G)GGGCG.

The protein in the DNA-binding domain EGR1 is a nuclear protein and functions as a transcriptional regulator. The products of the target genes that it activates are required for differentiation and mitogenesis. When located in the brain, it has an essential role in memory formation and in brain neuron epigenetic reprogramming. An X-ray crystal structure is available in the protein data bank as PDB: 4R2A. In such a EGR1 DNA-binding domain, the DNA chains are rel = AGCGTGGGCGT and rel = TACGCCCACGC.

As for the Fos domain above, let us consider the group $fp=\langle A,T,G,C|\mathrm{rel})\rangle$ on 4 letters with the relation bind or rel. The card seq of fp up to index 6 is similar to that of the free group $F_{3}=\langle A,T,G,C|\mathrm{ATGC})\rangle$ of rank 3. One can use the coset enumeration (with the Todd–Coxeter procedure) to check that, up to index 6, the permutation groups organizing the cosets in the cc of subgroups of $f_{p}$ and $F_{3}$ are the same. This shows that both groups are close, at least in the finite range of subgroups. However, fp and $F_{3}$ are not the same group. Again, the groups built from the joint relations of fp and $F_{3}$ are of rank 2, but the cardinality structure of cc of subgroups is not that of $F_{2}$.

The group $fp^{\prime }=\langle A,T,G,C|\mathrm{ATGC},\mathrm{bind})\rangle$, with the joint relations of fp and $F_{3}$, is close to the free group $F_{2}=\langle x,y,z|xyz\rangle$ on two generators in the sense that the card seq is that of $F_{2}$, up to the higher index 9 that we could reach in our calculations.

The early growth response protein 1 contains the chain of amino acids

GPLGS ERPYACPVESCDRRFSRSDELTRHIRIHTG QKPFQCRICMRNFSRSDHLTTHIRTHTG EKPFACDICGRKFARSDERKRHTKIHLR QKD.

The central portion of the protein contains 86=30+28+28 aa decomposed into 3 zinc fingers with the following secondary structure (letter H is for the α-helix segment, letter E is for the β-sheet segment and letter C is for the random coil segment)

CCCEECCCCCCCCEECHHHHHHHHHHHHHH CCCEECCCCCCEECHHHHHHHHHHHHHH CCCEECCCCCCEECHHHHHHHHHHHHHC

Taking the former 3-letter chain as the relation of a finitely generated group on 3 letters (and rank 2), we get the cardinality sequence for the cc of its subgroups as [1,3,7,26,112,717,⋯], which fits the cardinality sequence of cc of subgroups of the free group $F_{2}$ only up to the index 4.

#### 3.4.4. The DNA-Binding Domain Myc

**Figure 3:**
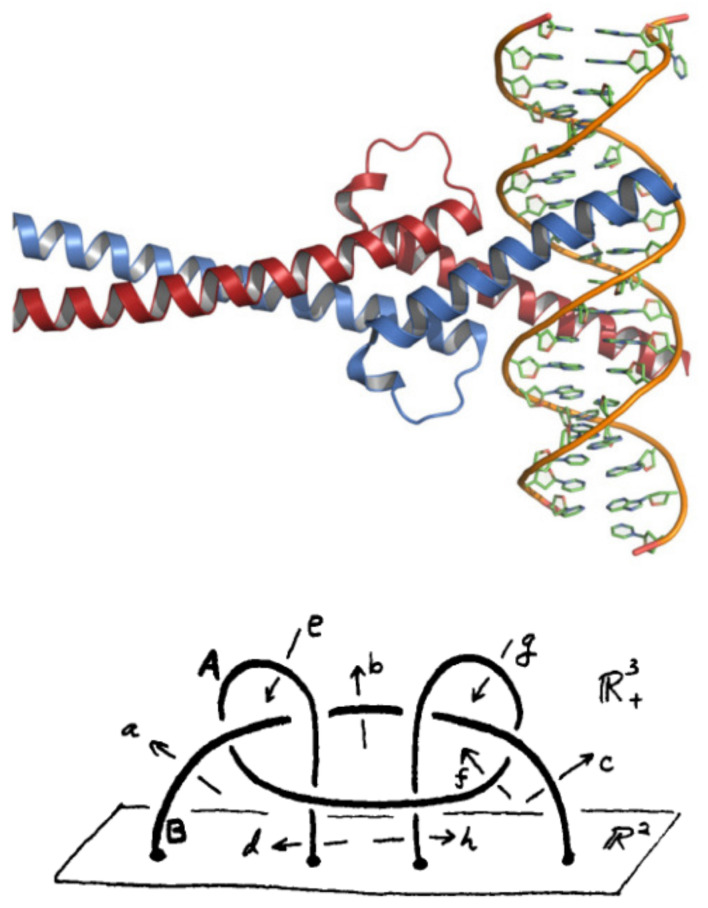
(**Up**) Crystal structure of Myc and Max in complex with DNA. (**Down**) The link L=A∪B (which is supposed to control the binding domain Myc) is attached to the plane $R^{2}$ in the half-space $R_{+}^{3}$. It is not splittable. This can be proven by checking that the fundamental group $\pi =\pi_{2}\left(L\right)$ is not free [22] and ([23] p. 90). One gets $\pi_{2}=\langle x,y,z|(x,(y,z))=z\rangle$, where (.,.) means the group theoretical commutator. The cardinality sequence of cc of subgroups of $\pi_{2}$ is [1,3,10,51,164,1365,9422,81594,721305,⋯].

Myc proto-oncogene is a transcription factor encoding a nuclear phosphoprotein that plays a role in cell cycle progression, apoptosis and cellular transformation [21]. The protein contains a basic helix-loop-helix zipper (bHLHZ) structural motif. The encoded protein forms a heterodimer with the related transcription factor Max, as shown in Figure 3. Amplification of this gene is frequently observed in numerous human cancers. Translocations involving this gene are associated with Burkitt lymphoma and multiple myeloma in human patients.

The bHLHZ domain of Myc-Max binds to the common DNA (palindromic) target 5${}^{\prime }$-CACGTG-3${}^{\prime }$. In the protein data bank, the reference of the complex is PDB: 1NKP. The whole DNA chain is

rel=CGAGTAGCACGTGCTACTC(1NKP_1).

Let us consider the group $fp=\langle A,T,G,C|\mathrm{rel})\rangle$ on 4 letters with the relation rel=bind=CACGTG. The conjugacy classes of fp up to index 6 have card seq equal to that of the free group $F_{3}=\langle A,T,G,C|\mathrm{GACT})\rangle$ of rank 3. Again, one can use the coset enumeration (with the Todd–Coxeter procedure) to check that, up to index 6, the permutation groups organizing the cosets in the cc of groups $f_{p}$ and $F_{3}$ are the same. This shows that both groups are close, at least in the finite range of subgroups. However, fp and $F_{3}$ are not the same group. The group $fp^{\prime }=\langle A,T,G,C|\mathrm{GACT},\mathrm{bind})\rangle$, with the joint relations of fp and $F_{3}$, is close to the group $\pi_{2}$ defined in Figure 3 (Down). The group $\pi_{2}=\langle x,y,z|(x,(y,z))=z\rangle$ is the fundamental group of the union of two links *A* and *B*, which are not splittable. The proof is in Refs. [22] and ([23] p. 90) and follows from the fact that $\pi_{2}$ is not a free group. The group $fp^{\prime }$ is close to $\pi_{2}$ in the sense that the cardinality sequence [1,3,10,51,164,1365,9422,81594,721305,⋯] of the cc of subgroups is that of $\pi_{2}$, up to the higher index 9 that we could reach in our calculations.

The non-closeness of $fp^{\prime }$ to $F_{2}$ and the fact that $\pi_{2}$ is not free are distinguishing features of the Myc domain. It is tempting to associate such features with a potential abnormal replication.

### 3.5. Genes Whose Transcription Factors Have a Group Structure Away from a Free Group

We analyzed the group structure of motifs for some transcription factors that do not lead to free groups. This is shown in Table 5. A short account of the function or dysfunction of the corresponding genes is given in Table 6. It is observed that several transcription factors whose group structure is away from a free group have the same card seq. We conjecture that it is indicative of a related 3-dimensional structure of the corresponding domain, although these families do not fit the standard classification [6].

#### The DNA-Binding Domain of p53

Tumor protein p53 (also called tumor suppressor p53) has been called the guardian of the genome. The main reason behind this status is the critical role that p53 plays in preventing cancer development. p53’s role in tumor suppression is due to its ability to induce the apoptosis, cell cycle arrest and senescence of pre-cancerous cells. However, it also regulates other genes involved in metabolism.

According to [25], a motif for p53 is the DNA sequence CACATGTCCA. In our Table 5, the attached card seq in the finite range of indices is that of a group $\pi_{3}^{\prime }$. However, there are motifs leading to a card seq associated with the free group $F_{3}$ or with other non-free groups that are not of type $\pi_{3}^{\prime }$. This may be due to the fact that p53 has many isoforms to fill its role.

**Figure 4:**
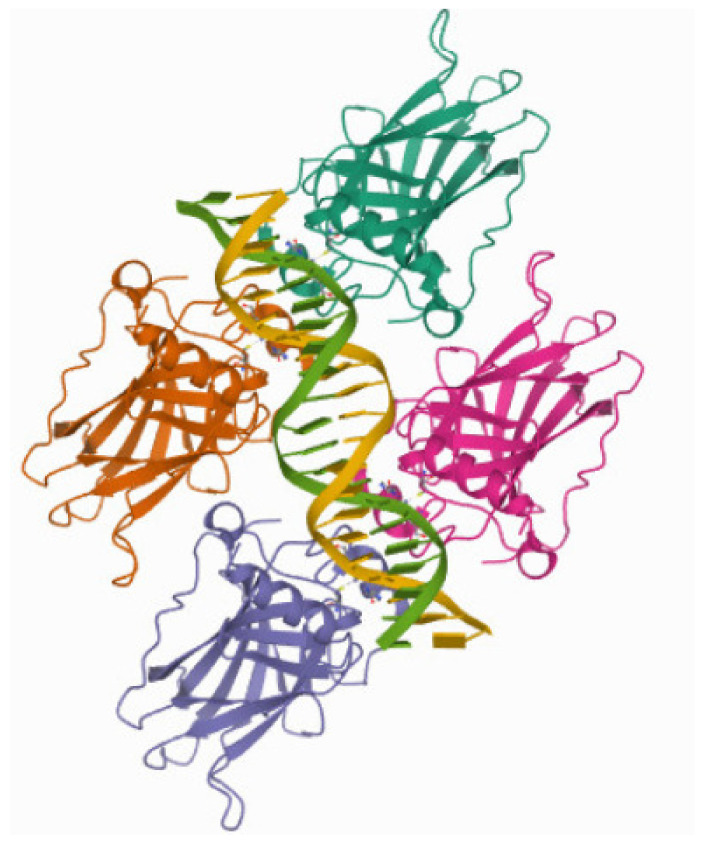
Crystal structure of p53 binding domain. The reference number in the protein data bank is 4HJE.

In Figure 4, we draw the crystal structure of the p53 domain for the binding domain of the PDB sequence 4HJE [27]. The p53 domain forms a tetramer, but other symmetries of the binding domain of p53 may be found.

## 4. Discussion

According to Reference [1], aperiodicity is the correlate of syntactical freedom of ordering rules. How can we check this statement in the realm of transcription factors?

First, we introduce the concept of a general substitution rule in the context of free groups. A general substitution rule ρ on a finite alphabet $A_{r}$ on *r* letters is an endomorphism of the corresponding free group $F_{r}$[28] (Definition 4.1). The endomorphism property means the two relations ρ(uv)=ρ(u)ρ(v) and $\rho \left(u^{-1}\right)=\rho^{-1}\left(u\right)$, for any $u,v\in F_{r}$.

A special role is played by the subgroup $\mathrm{Aut}(F_{r})$ of automorphisms of $F_{r}$. We introduce the map $\alpha :F_{r}\to Z_{r}$ from $F_{r}$ to the Abelian group $Z_{r}$ in order to investigate the substitution rule ρ with the tools of matrix algebra.

The map α induces a homomorphism $M:\mathrm{End}\left(F_{r}\right)\to \mathrm{Mat}\left(r,Z\right)$. Under *M*, $\mathrm{Aut}(F_{r})$ maps to the general linear group of matrices with integer entries GL(r,Z). Given ρ, there is a unique mapping M(ρ) that makes the map diagram commutative [28] (p. 68). The substitution matrix M(ρ) of ρ may be specified by its elements at row *i* and column *j* as follows

$${\left(M\left(\rho \right)\right)}_{i,j}=\mathrm{card}\left(\rho_{a_{i}}\left(a_{j}\right)\right).$$

We will apply this approach to binding motifs of transcription factors. The binding motif rel in the finitely presented group $fp=\langle A,T,G,C|\mathrm{rel}(A,T,G,C)\rangle$ is split into appropriate segments so that $\mathrm{rel}={\mathrm{rel}}_{A}{\mathrm{rel}}_{T}{\mathrm{rel}}_{G}{\mathrm{rel}}_{C}$ with the substitution rules $A\to rel_{A}$, $T\to rel_{T}$, $G\to rel_{G}$, $C\to rel_{C}$.

We are interested in the sequence of finitely generated groups

$$f_{p}^{\left(l\right)}=\langle A,T,G,C|\mathrm{rel}(\mathrm{rel}(\mathrm{rel}\cdots (A,T,G,C)))\rangle \;\;\left(\mathrm{with}\;\mathrm{rel}\;\mathrm{applied}\;l\;\mathrm{times}\right)$$

whose card seq is the same at each step *l* and equal to the card seq of the free group $F_{r}$ (in the finite range of indices that it is possible to check with the computer).

Under these conditions, we will see that (group) syntactical freedom correlates to the aperiodicity of sequences.

### 4.1. Aperiodicity of Substitutions

There is no definitive classification of aperiodic order, the intermediate between crystalline order and strong disorder, but in the context of substitution rules, some criteria can be found. We need a few definitions.

A non-negative matrix M∈Mat(d,R) is one whose entries are non-negative numbers. A positive matrix *M* (denoted M>0) has at least one positive entry. A strictly positive matrix (denoted M>>0) has all positive entries. An irreducible matrix $M={\left(M_{ij}\right)}_{1\leq i,j\leq d}$ is one for which there exists a non-negative integer *k* with ${\left(M^{k}\right)}_{ij}>0$ for each pair (i,j). A primitive matrix *M* is one such that $M^{k}$ is a strictly positive matrix for some *k*.

A Perron–Frobenius (PF for short) eigenvector *v* of an irreducible non-negative matrix is the only one whose entries are positive: v>0. The corresponding eigenvalue is called the PF eigenvalue.

We will use the following criterion [28] (Corollary 4.3). A primitive substitution rule ρ of substitution matrix M(ρ) with an irrational PF-eigenvalue is aperiodic.

A well-studied primitive substitution rule is the Fibonacci rule $\rho =\rho_{F}:\;a\to ab,\;b\to a$ of substitution matrix $M_{F}=\left(\begin{array}{cc} 1 & 1\\ 1 & 0 \end{array}\right)$ and PF eigenvalue equal to the golden ratio $\lambda_{PF}=\tau =\left(\sqrt{5}+1\right)/2$[28] (Example 4.6). As expected, the irrationality of λ corresponds to the aperiodicity of the Fibonacci sequence.

The sequence of Fibonacci words is as follows:

a,b,ab,aba,abaab,abaababa,abaababaabaab,⋯

The words have lengths equal to the Fibonacci numbers 1,1,2,3,5,8,13,21,⋯

It is straightforward to check that all finitely generated groups $f_{p}^{\left(l\right)}$ whose relations rel(a,b)=ab,aba,abaab,abaababa,⋯ have a card seq whose elements are 1’s, as for the card seq of the free group $F_{1}$. The Fibonacci sequence is our first example where group syntactical freedom correlates to aperiodicity.

We will now provide examples taken for transcription factors involving 2, 3 or 4 letters.

#### 4.1.1. A Two-Letter Sequence for the Transcription Factor of Gene DBX in Drosophila Melanogaster

Let us consider the motif rel = TTTATTA for the gene DBX in drosophila melanogaster (fruit flies) [6] (MA0174.1). The roles of the DBX gene include neuronal specification and differentiation.

We split rel into two segments so that $\mathrm{rel}={\mathrm{rel}}_{A}{\mathrm{rel}}_{T}$ with the substitution maps $A\to {\mathrm{rel}}_{A}=TTTA$, $T\to {\mathrm{rel}}_{T}=TTA$ to produce the substitution sequence

A,T,AT,TTTATTA,TTATTATTATTTATTATTATTTA⋯

The substitution matrix for this sequence is $M=\left(\begin{array}{cc} 1 & 3\\ 1 & 2 \end{array}\right)$; it is a primitive matrix of PF eigenvalue $\lambda_{PF}=\left(3+\sqrt{13}\right)/2$ so that the sequence associated with the DBX factor is aperiodic.

Similarly to the Fibonacci generator rules, all finitely generated groups $f_{p}^{\left(l\right)}$ whose relations are rel(A,T)=AT,TTTATTA,TTATTATTATTTATTATTATTTA⋯ have a card seq whose elements are 1’s as for the card seq of the free group $F_{1}$.

The sequence for the DBX transcription factor is our second example where group syntactical freedom correlates to aperiodicity.

#### 4.1.2. A Three-Letter Sequence for the Transcription Factor of Gene EGR1

The transcription factor of gene EGR1 was investigated in Section 3.4.1. The selected motif is rel = GCGTGGGCG. We split rel into two segments so that $\mathrm{rel}={\mathrm{rel}}_{C}{\mathrm{rel}}_{G}{\mathrm{rel}}_{T}$ with the substitution maps $C\to {\mathrm{rel}}_{C}=G$, $G\to {\mathrm{rel}}_{G}=CGT$, $T\to {\mathrm{rel}}_{T}=GGGCG$ to produce the substitution sequence:

C,G,T,CGT,GCGTGGGCG,CGTGCGTGGGCGCGTCGTCGTGCGT⋯

The substitution matrix for this sequence is $M=\left(\begin{array}{ccc} 0 & 1 & 1\\ 1 & 1 & 4\\ 0 & 1 & 0 \end{array}\right)$; it is a primitive matrix (since $M^{2}>>0$) whose eigenvalues follow from the vanishing of the polynomial $-\lambda^{3}+\lambda^{2}+5\lambda +1=0$. There are three real irrational roots $\lambda_{1}\approx 2.86619$, $\lambda_{2}\approx -0.21075$ a,d $\lambda_{3}\approx -1.65544$. The PF eigenvalue is $\lambda_{PF}=\lambda_{1}$ with an eigenvector of (positive) entries $(1,\lambda_{1}/\left(\lambda_{1}^{2}-\lambda_{1}-4\right),1/{\left(\lambda_{1}^{2}-\lambda_{1}-4\right)}^{T}\approx {\left(1,2.12485,0.74134\right)}^{T}$.

It follows that the selected sequence for the EGR1 gene is aperiodic.

All finitely generated groups $f_{p}^{\left(l\right)}$ whose relations are rel(C,G,T)=CGT,GCGTG *GGCG*,CGTGCGTGGGCGCGTCGTCGTGCGT⋯ have a card seq whose elements are those of the free group $F_{2}$.

The sequence for the EGR1 transcription factor is our third example where group syntactical freedom correlates to aperiodicity.

#### 4.1.3. A Four-Letter Sequence for the Transcription Factor of the Fos Gene

The transcription factor of gene Fos was investigated in Section 3.4.1. The selected motif is rel = TGAGTCA.

We split rel into two segments so that $\mathrm{rel}={\mathrm{rel}}_{A}{\mathrm{rel}}_{T}{\mathrm{rel}}_{G}{\mathrm{rel}}_{C}$ with the substitution maps $A\to {\mathrm{rel}}_{A}=T$, $T\to {\mathrm{rel}}_{T}=G$, $G\to {\mathrm{rel}}_{G}=AGTC$, $C\to {\mathrm{rel}}_{C}=A$, to produce the substitution sequence

A,T,G,C,ATGC,TGAGTCA,GAGTCTAGTCGAT⋯

The substitution matrix for this sequence is $M=\left(\begin{array}{cccc} 0 & 0 & 1 & 1\\ 1 & 0 & 1 & 0\\ 0 & 1 & 1 & 0\\ 0 & 0 & 1 & 0 \end{array}\right)$. It is a primitive matrix ($M^{4}>>0$) whose eigenvalues follow from the vanishing of the polynomial $\lambda^{4}-\lambda^{3}-\lambda^{2}-\lambda -1$. There are two real eigenvalues $\lambda_{1}\approx 1.92756$ and $\lambda_{2}\approx -0.77480$, as well as two complex conjugate eigenvalues $\lambda_{3,4}\approx -0.07637\pm 0.81470i$.

The PF eigenvalue is $\lambda_{PF}=\lambda_{1}$ with an eigenvector of (positive) entries ≈(1,0.37298,0.40211, 0.20861 )${}^{T}$.

It follows that the selected sequence for the Fos gene is aperiodic.

All the finitely generated groups $f_{p}^{\left(l\right)}$ whose relations are:

$$\begin{array}{cc} \; & \mathrm{rel}\left(A,C,G,T\right)\;=\;\mathrm{ATGC},\;\mathrm{TGAGTCA},\;\mathrm{GAGTCTAGTCGAT}\cdots \\ \; & \mathrm{have}\;a\;\mathrm{card}\;\mathrm{seq}\;\mathrm{whose}\;\mathrm{elements}\;\mathrm{are}\;1,\;7,\;41,\;604,\;13753,\;504243\cdots . \end{array}$$

which is the card seq of the free group $F_{3}$.

The sequence for the Fos transcription factor is our fourth example where group syntactical freedom correlates to aperiodicity.

## 5. Conclusions

We made use of group theory for investigating transcription factors in genetics. Finite group theory plays a major role in the attempts to model the genetic code; see [29,30] and other references therein. Finitely generated groups (whose cardinality is infinite) are necessary to deal with the secondary structures of proteins [2]. It was already noted that many structures for the protein secondary codes tend to be close to free groups. The card seq for such codes is model-dependent. In the map from amino acids to proteins, the transcription factors play a critical role. The study of the group theoretical structure of TFs has been our goal in the present paper. The DNA motifs that serve as a relation for the corresponding fp groups are, in general, short sequences with around 10 amino acids. Taking random sequences instead of the gene-specific DNA sequences in TFs also leads to a majority of cases where the card seq of fp is close to a free group $F_{r}$ and less frequent cases where the card seq is away. However, motifs in TFs are codes with a particular meaning—the specific gene function or dysfunction. In this sense, we found it appropriate to use the concept of ‘syntactical freedom’ to qualify most TFs and to associate the lack of syntactical freedom with a potential source of illness. In our context, syntactical freedom means free groups and aperiodicity.

Potentially, there are many potential applications of our group theoretical method of TFs for characterizing genes with problematic mutations, such as cancer and Alzheimer’s disease. More work will be performed in the future.

Another interesting line of research is about the neurogenetic correlation of human consciousness and the related TFs. If the reader is interested in studying this research further, we recommend [24,31,32,33].

As a final note, we refer to the paper [34,35] in the domain of quantum gravity, where the ordering rules with syntactical freedom are those of quasicrystals instead of those of the biological crystal structures.

## Figures and Tables

**Table 1:** The number of conjugacy classes of subgroups of index *d* in the free group of rank *r* [9].

r	d = 1	d = 2	d = 3	d = 4	d = 5	d = 6	d = 7
1	1	1	1	1	1	1	1
2	1	3	7	26	97	624	4163
3	1	7	41	604	13,753	504,243	24,824,785
4	1	15	235	14,120	1,712,845	371,515,454	127,635,996,839
5	1	31	1361	334,576	207,009,649	268,530,771,271	644,969,015,852,641

**Table 2:** Group structure of a TATA box. Column 1 is for the selected consensus sequence (rows 4 to 6 are for the TATA box in the core promoter of UGTIA1 gene). Column 2 is for the cardinality sequence (card seq) of conjugacy classes (cc) of subgroups in the finitely generated group whose relation (rel) is the consensus sequence (cons seq). Column 3 identifies the Hecke group $H_{q}=\langle A,T|A^{2}T^{-q}\rangle$, which is close to the group under consideration (based on its card seq of subgroups). Column 4 refers to some references in the literature. Bold digits feature the fit to a Hecke group.

Rel: Cons Seq	Card Struct of cc of Subgroups	Group	Literature
TATAAAA	[1,1,2,3,2,8,7,10,18,28,⋯]	$H_{3}$	[6] (MA0108.1)
TATAAAAA	[1,3,2,8,6,19,16,69,83,238,⋯]	$H_{4}$	[13]
A(TA)${}_{5}$TAA	[1,3,3,7,6,34,42,123,319,706,⋯]	$H_{6}$	[14]
A(TA)${}_{6}$TAA	[1,1,1,1,1,1,34,77,79,51,⋯]	$H_{7}$	.
A(TA)${}_{7}$TAA	[1,3,2,8,6,19,16,171,315,1022⋯]	$\approx H_{4}$	.
A(TA)${}_{8}$TAA	[1,1,2,3,2,8,7,10,308,792⋯]	$\approx H_{3}$	.

**Table 3:** Group analysis of a few known and candidate SNP markers (taken from [15]) Column 1 is for the selected gene. Column 2 is for the SNP marker. Column 3 is for the card seq for the finitely generated group fp whose relation (rel) is the marker. Column 4 is for the reference paper and the letter indicates the heuristic confidence level of the candidate SNP marker (in alphabetical order from the best (A) to the worst (E)). The computed closeness of the finitely generated group to the free group $F_{2}$, most of time, correlates to a lower risk of illness, as described in [15]. The symbol * corresponds to the only two-base SNP marker in the table. The card seq is the same as the sequence for the fundamental group of 3-manifold m002. The latter manifold is the smallest volume closed 3-manifold and is non-orientable [17].

Gene	Rel: Marker	Card Seq of cc of Subgroups	Literature
ESR2	TTAAAAGGAA	[1,7,17,114,423,4526,30364,293306⋯]	Table 1 in [15], B
HSD17B1	AGCCCAGAGC	[1,3,7,26,217,124,18443,219870⋯]	., A
.	CAAGCCCAGA	[1,7,14,109,396,3347,19758,188940⋯]	., A
PGR	AAAGGAGCCG	[1,7,17,142,475,4125,23509,225871⋯]	., A
GSTM3	GGGTATAAAG	[1,7,14,109,396,3347,19758,188940⋯]	., E
.	CCCCTCCCGC	[1,3,7,26,97,624,4163,34470⋯]	., C
.	CCCTCCCGCT	.	., C
IL1B	AAAACAGCGA	[1,7,14,89,224,1842,10191,86701⋯]	Table 2 in [15], A
CYP2A6	AAAGGCAAC	[1,7,17,134,683,7077,64225⋯]	., A
DHFR	GGGACGAGGG	[1,3,7,26,97,624,4163,34470⋯]	., A
.	GGACGAGGGG	.	., A
LEP	GGGGCGGGA	[1,3,7,26,97,624,4163,34470⋯]	Table 3 in [15], C
GCG	TGCGCCTTGG	[1,3,7,26,119,816,4865,40489⋯]	., B
GH1	TATAAAAAGG	[1,7,14,109,396,3347,19758,188940]	., E
.	GTATAAAAAG	.	., D
.	GGTATAAAAA	.	., E
.	AGGGCCCACA	[1,3,7,26,127,860,5661,45710⋯]	., A
.	AAAGGGCCCC	[1,3,10,67,266,3458,30653,312237⋯]	., A
.	AAAGGGCCA	.	., A
NOS2	TCTTGGCTGC	[1,3,7,26,97,624,4163,34470⋯]	Table 4 in [15], A
TPI1	ATATAAGTGG	[1,3,7,30,125,856,4832,40246⋯]	., B
GJA5	TATTAAACAC	[1,3,10,35,140,921,5778,47238⋯]	., E
HBD	AAAAGGCAGG	[1,3,7,26,97,624,4163,34470⋯]	Table 5 in [15], A
F2	AACCCAGAGG	[1,3,7,26,127,860,5661,45710⋯]	., A
F8	GGAAGAGGGA	[1,3,2,7,4,18,9,27,36,68⋯] *	Table 6 in [15], A
F3	GCGCGGGGCA	[1,3,7,26,97,624,4163,34470⋯]	., A
F11	TTTTTAGTAA	.	., D
.	TTTTTAGTAA	[1,7,17,114,423,4526,30364,293306⋯]	., A
.	AAGGAAATTT	[1,3,7,26,195,1692,11803,73192⋯]	., A
AR	GTGGAAGATT	[1,3,7,34,139,931,5208,43867⋯]	Table 7 in [15], A
.	CCACGACCCG	[1,7,20,167,754,7232,60860,683597⋯]	., D
MTHFR	TCCCTCCCA	[1,3,7,26,97,624,4163,34470⋯]	., A
DMNT1	TGTGTGGCCCG	.	., A
.	GTGTGTGCCC	.	., A
.	GACGAGCCCA	[1,3,7,42,131,912,6011,47322⋯]	., A
NR5A1	ACAAGAGAAA	[1,3,7,26,97,624,4163,34470⋯]	., A
.	GGTGTGAGAG	[1,7,14,89,264,1987,11086,93086⋯]	., A

**Table 4:** Group structure of motifs for transcription factors of immediately early genes Fos, EGR and Myc. Most of the time, the card seq of the group defined with the relation/motif is the free group $F_{2}$ (for a 3 letter motif) or $F_{3}$ (for a 4 letter motif). There are two exceptions for the EGR1 gene, depending on the selected motif, where the card seq corresponds to the modular group $H_{3}$ or the Baumslag–Solitar group BS(−1,1), which is the fundamental group of the Klein bottle. The card seq for $H_{3}$ is in Table 2. The card seq for BS(−1,1) is [1,3,2,5,2,7,2,8,3,8,2,13,2,9,4,⋯].

Gene	Rel: Motif	Card Seq	Literature
Fos	TGAGTCA	$F_{3}$	[19]
.	TGACTCA	$F_{3}$	[6], MA MA0099.2
EGR1	GCGTGGGCG	$F_{2}$	[6], MA0162.1
.	CCGCCCCCG	$H_{3}$	., MA0162.2
.	CCGCCCCCGC	BS(−1,1)	., .
.	ACGCCCACGCA	$F_{2}$	., MA0162.3
.	GGCCCACGC	.	., MA0162.4
EGR2	CCGCCCACGC	.	., MA0472.1
.	ACGCCCACGCA	.	., MA0472.2
EGR3,EGR4	ACGCCCACGCA	.	., [ MA0732.1, MA0733.1]
Myc	CACGTG	$F_{3}$	[19]
.	CGCACGTGGT	.	[6], MA0147.1
.	CCCACGTGCTT	.	., MA0147.2
.	CCACGTGC	.	., MA0147.3
Mycn, Max::Myc, etc	GACCACGTGGT, etc.	.	., [MA0104.1, etc.]

**Table 5:** Group structure of motifs for some transcription factors that are not leading to free groups. The card seq for $\pi_{1}$ is [1,4,1,2,4,2,1,7,2,2,4,2,2,8,1,2,7,2,3,⋯]; for $\pi_{1}^{\prime }$ it is [1,1,1,2,1,3,3,1,2,2,1,1,9,2,14,2,1,⋯]. The card seq for $\pi_{2}$ is already in Figure 3 as [1,3,10,51,164,1365,9422,81594,721305,⋯]. The card seq for $\pi_{3}$ is [1,7,14,89,264,1987,11086,93086,⋯]; for $\pi_{3}^{\prime }$, it is [1,7,50,867,15906,570528,⋯]; for $\pi_{3}^{\prime \prime }$, it is [1,7,50,739,15234,548439,⋯]; for $\pi_{3}^{\left(3\right)}$, it is [1,7,41,668,14969,550675]. The card seq for $\pi_{4}$ is [15,82,1583,30242⋯]. The index *i* in $\pi_{i}$ refers to the rank of the group under examination. The three sections are for motifs on 2, 3 and 4 letters, respectively.

Gene	Rel: Motif	Card Seq	Literature
NKX6-2	TAATTAA	$H_{3}$	[6], [MA0675.1, MA0675.2]
HoxA1, HoxA2	TAATTA	$\pi_{1}$	[6], [MA1495.1, MA0900.1]
POU6F1, Vax	.	.	., [MAO628.1, MA0722.1]
RUNX1	TGTGGT	.	., MA0511.1
RUNX1	TGTGGTT	$\pi_{1}^{\prime }$	[6], MA0002.2
EHF	CCTTCCTC	.	., MA0598.1
POU6F1	TAATGAG	$\pi_{2}$	[6] MA1549.1
PITX2	TAATCCC	.	., [MA1547.1, MA1547.2]
ELK4	CTTCCGG	.	., MA0076.2
OTX2, Dmbx1	GGATTA	$\pi_{3}$	[6], [MA0712.2, MA0883.1]
PitX1, PitX2, PitX3, OTX1	TAATCC	.	.,[MA0682.1, MA0711.1]
N-box	TTCCGG	.	[24]
p53	CACATGTCCA	$\pi_{3}^{\prime }$	[25]
GZF1	TGCGCGTCTATA	.	[4]
NF-kappa-B	GGGAATTTCC	.	[6], [MA0107.1, MA1911.1]
STAT1	TTTCCCGGAA	.	., MA0137.2
.	TTCCAGGAA	.	., MA0137.3
STAT4	TTCCAGGAAA	.	., MA0518.1
FOSL1::Jun	ATGACGTCAT	$\pi_{3}^{\prime \prime }$	[6], MA1129.1
USF2	GTCATGTGACC	.	. , MA0626.1
PAX1	CGTCACGCATGA	.	. , MA0779.1
STAT2	TTCCAGGAAG	.	. , MA0144.1
FOS	GATGACGTCATCA	$\pi_{3}^{\left(3\right)}$	[6], MA1951.1
MAFA, MAFF,MAFK	TGCTGAGTCAGCA	.	., [MA1521.1, MA0495.2, MA0946.2]
CREB	TGACGTCA	$\pi_{4}$	[6], [MA0018.2, MA018.3]
USF2	GGTCACGTGACC	.	., MA0526.4
SMAD3, SMAD5	GTCTAGAC	.	., [MA0795.1, MA1557.1], [26]

**Table 6:** A short account of the function or dysfunction (through mutations or isoforms) of genes associated with transcription factors and sections in Table 5.

Gene	Type	Function	Dysfunction
NKX6-2	homeobox	central nervous system, pancreas	spastic ataxia
HoxA1	homeobox	embryonic devt of face and heart	autism
HoxA2	.	.	cleft palate
Pou6F1	.	neuroendocrine system	clear cell adenocarcinoma
Vax	.	forebrain development	craniofacial malform.
RunX1	Runt-related	cell differentiation, pain neurons	myeloid leukemia
EHF	homeobox	epithelial expression	carcinogenesis, asthma
PitX2	.	eye, tooth, abdominal organs	Axenfeld–Rieger syndrome
ELK4	Ets-related	serum response for c-Fos	
OTX1,OTX2	homeobox	brain and sensory organ devt	medulloblastomas
Dmbx1	.	.	farsightedness and strabismus
PitX1	.	organ devt, left–right asymmetry	autism, club foot
PitX3	.	lens formation in eye	congenital cataracts
N-box	Ets-related	synaptic expression	drug sensitivity
p53	p53 domain	‘Guardian of the genome’	cancers
GZF1	Zinc fingers	protein coding	short stature, myopia
NF-kappa-B	.	DNA transcription, cytokines	apoptosis
STAT1	Stat family	signal activator of transcription	immunodeficiency 31
STAT4	Stat family	signal activator of transcription	rheumatoid arthritis
FOSL1::Jun	leucine zipper	cellular proliferation	marker of cancer
USF2	helix-loop-helix	transcription activator	
PAX1	paired box	fetal development	Klippel–Feil syndrome
FOS	leucine zipper	cellular proliferation	cancers
Maf	.	pancreatic development	congenital cerulean cataract
CREB	bZIP	neuronal plasticity	Alzheimer’s disease
USF2	helix-loop-helix	transcription activator	
SMAD	homeo domain	cell development and growth	Alzheimer’s disease

## Data Availability

Computational data are available from the authors.
